# Coexistence of CSF anti-Ma2 antibody and 14-3-3 protein: a diagnostic dilemma between autoimmune encephalitis and Creutzfeldt-Jakob disease, a Case Report

**DOI:** 10.3389/fimmu.2025.1598626

**Published:** 2025-08-08

**Authors:** Nianlong Sun, Liangyu Zou, Jian Deng, Fengqing Wei, Hui Zhang

**Affiliations:** ^1^ Department of Radiology, People’s Hospital of Baoan Shenzhen, Shenzhen, Guangdong, China; ^2^ Department of Neurology, The Second Clinical Medical College, Jinan University, Shenzhen, Guangdong, China; ^3^ Department of Neurology, The First Affiliated Hospital (Shenzhen People’s Hospital), Southern University of Science and Technology, Shenzhen, Guangdong, China; ^4^ Department of Neurology, Shenzhen Longgang Second People’s Hospital, Shenzhen, Guangdong, China

**Keywords:** anti-Ma2 antibody, autoimmune encephalitis (AE), 14-3-3 protein, Creutzfeldt-Jakob disease, case report

## Abstract

**Background:**

Anti-Ma2 antibody encephalitis is a rare paraneoplastic autoimmune encephalitis (AE) caused by anti-Ma2 antibody. Creutzfeldt-Jakob disease (CJD), a group of human prion diseases, is a rapidly advancing and fatal neurodegenerative disorder. The two diseases may display comparable clinical symptoms that are easily misdiagnosed. The 14-3–3 protein in the Cerebrospinal fluid (CSF) is considered a valuable marker for diagnosing CJD. In this report, we discussed a case of anti-Ma2 antibody encephalitis in which the CSF showed positive results for 14-3–3 protein, a new instance of antibody coexistence.

**Case presentation:**

A 77-year-old man was hospitalized due to his recent rapid progression of memory loss, mental and behavioral abnormalities, and gait disturbance. Brain CT showed no abnormalities. The detection of antineuronal antibodies in serum and CSF using Western blot revealed positive high titers for anti-Ma2 antibody. Surprisingly, the 14-3–3 protein in CSF was positive. Subsequently, FLAIR magnetic resonance imaging showed abnormal regions with heightened signal intensity in the bilateral mesial temporal lobes, amygdala, and hippocampus. Electroencephalography, real-time quaking-induced conversion in CSF, and prion protein gene in blood were detected to distinguish from CJD, and these findings did not match the diagnosis of CJD. Finally, the patient was treated with intravenous methylprednisolone, intravenous immunoglobulin (IVIG), and rituximab. The patient’s condition was effectively improved.

**Conclusions:**

Anti-Ma2 antibody encephalitis is a type of encephalitis associated with autoantibodies targeting intracellular antigens. Previous studies have detected the presence of 14-3–3 protein in some cases of AE associated with antibodies against neuronal surface antigens. This is the first report of concomitant anti-Ma2 antibody and CSF 14-3–3 protein positivity, which is a further extension of previous studies. This case demonstrates that CSF 14-3–3 protein positivity does not preclude AE and may reflect secondary neuronal injury. When both antibodies are present simultaneously, the diagnosis should be made in combination with the patient’s imaging features, prion-specific testing and high titers of antineuronal antibodies to avoid delay in treatable disease.

## Introduction

Anti-Ma2 antibody encephalitis is a rare autoimmune encephalitis (AE) caused by anti-Ma2 antibody (1). When its initial clinical manifestations are cognitive impairment and mental and behavioral abnormalities, it is easily misdiagnosed as Creutzfeldt-Jakob disease (CJD). CJD, a group of human prion diseases, is a rapidly progressive and fatal neurodegenerative disorder with a median survival of 4–6 months (2). The 14-3–3 protein in cerebrospinal fluid (CSF) is considered to be a valuable marker for the diagnosis of CJD (3). Previous studies have demonstrated that the 14-3–3 protein can be detected in AE associated with antibodies against neuronal surface antigens (3, 4). However, we will report a case of anti-Ma2 antibody encephalitis targeting intracellular antigens with coexisting CSF 14-3–3 positivity, which is a new instance of antibody coexistence. To address the diagnostic challenges in differentiating anti-Ma2 antibody encephalitis from CJD when both antibodies coexist, this case aims to: (a) Characterize the clinico-radiological phenotype of anti-Ma2 encephalitis with coexisting 14-3–3 protein in CSF, (b) Systematically evaluate differential diagnoses through antibody testing (Western blot), FLAIR MRI, and prion-specific assays (RT-QuIC, PRNP analysis), and (c) Contextualize these findings within literature on antibody-coexisting neurological syndromes.

## Case presentation

**Figure 1:**
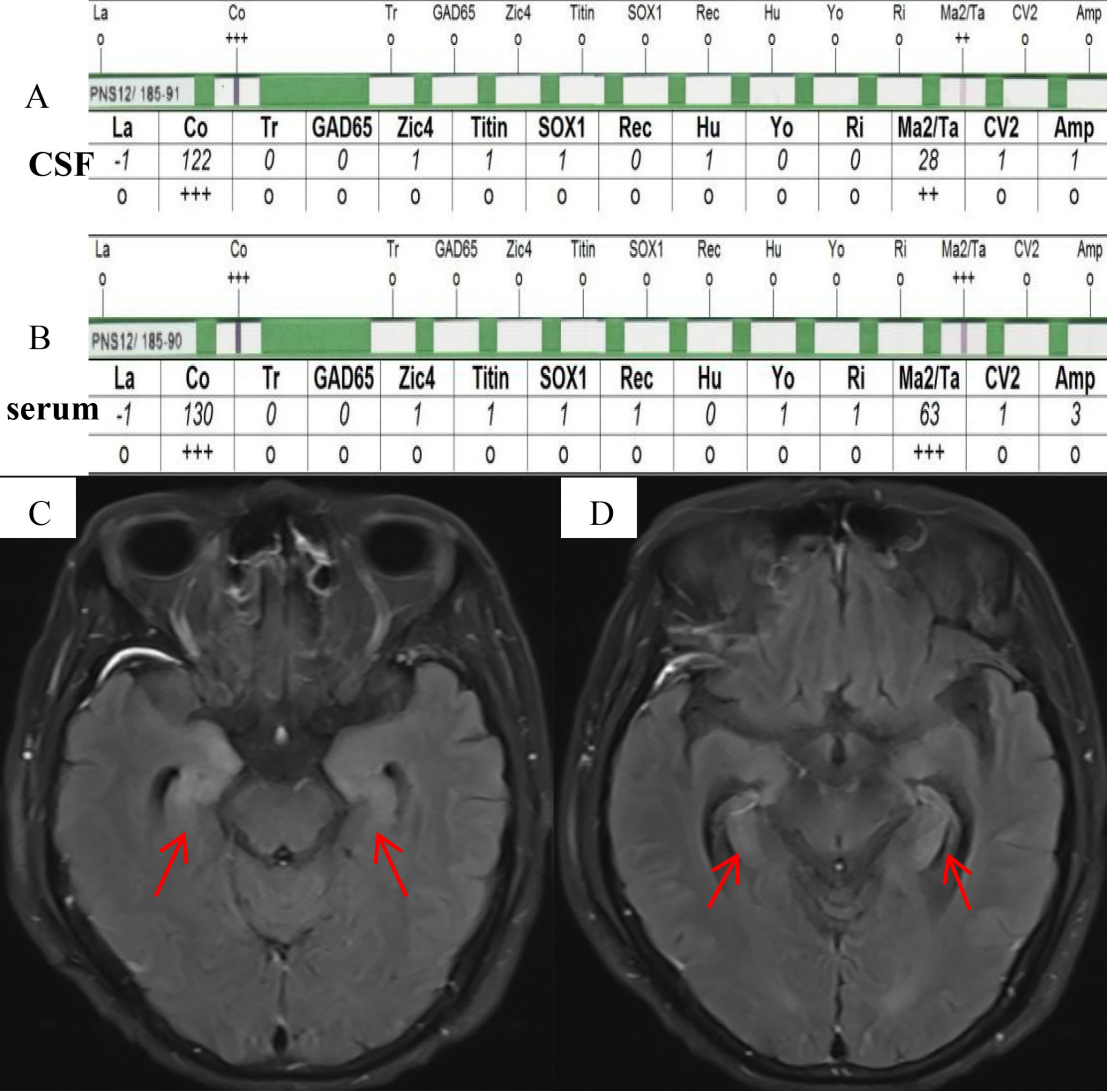
**(A, B)** Western blot analysis showed a positive anti-Ma2 antibody, corresponding to a high-intensity band in CSF **(C)** and a higher-intensity band in serum **(D)**. **(C, D)** FLAIR magnetic resonance imaging revealed abnormal regions characterized by elevated signal intensity in the bilateral mesial temporal lobes, amygdala, and hippocampus (red arrows).

A male patient, aged 77, was admitted to the hospital due to a prolonged period of 17 days characterized by symptoms including memory impairment, incoherent speech, hallucinations, and severe sleeplessness. He exhibited difficulty in opening both eyes, as well as a sluggish walking pace and frequent spitting of saliva. He had no prior history of infection such as fever. The results of the physical examination of the nervous system indicated a decline in memory, calculating, and orientation abilities, accompanied by ptosis, vertical gaze paralysis, and cogwheel rigidity in the extremities. Brain CT showed no abnormalities. Given the patient’s rapidly progressing cognitive impairment, mental abnormalities, and extrapyramidal symptoms, a lumbar puncture was performed to detect antibodies associated with autoimmune encephalitis and proteins associated with CJD. CSF biochemistry tests revealed no abnormalities. The presence of antineuronal antibodies associated with AE in the serum and CSF was identified through the utilization of indirect immunofluorescence and Western blot (WB) techniques using kits provided by EUROIMMUN AG, Germany. WB analysis revealed a positive result for anti-Ma2 antibody (anti-PNMA2 (Ma2/Ta) IgG++) in CSF (**Figure 1A**). In addition, strongly positive anti-Ma2 antibody was screened in the serum (anti-PNMA2 (Ma2/Ta) IgG+++, **Figure 1B**). Subsequently, FLAIR magnetic resonance imaging revealed abnormal regions characterized by elevated signal intensity in the bilateral medial temporal lobes, amygdala, and hippocampus (**Figures 1C, D**). No abnormalities were observed in the testes’ ultrasonography, and no lesions exhibiting heightened uptake on the whole-body 18F-fluorodeoxyglucose-PET/CT imaging were discovered. Consequently, the patient was diagnosed with paraneoplastic anti-Ma2 antibody encephalitis without a tumor. Surprisingly, the 14-3–3 protein in CSF was positive by Western blot at the Prion Disease Laboratory, China CDC. The electroencephalography analysis demonstrated diffuse slowing of the background activity with no periodic triphasic waves. Real-time quaking-induced conversion (RT-QuIc) in CSF and prion protein gene (PRNP) in blood were negative. The patient received intravenous methylprednisolone (500mg/day) and immunoglobulin (0.4g/kg/day) for five days, which resulted in a slight improvement in his symptoms. Subsequently, rituximab was administered two weeks later, a single dose of 600mg divided into two days, according to the expert consensus on the diagnosis and treatment of autoimmune encephalitis in China and the recommendations of previous studies (5, 6). His symptoms significantly improved. The oral prednisone therapy was maintained. At 3-month follow-up, the patient was able to take care of himself but still had cognitive impairment. Until six months after discharge, the patient died due to severe pneumonia.

## Discussion and conclusion

Anti-Ma2 antibody encephalitis is also a paraneoplastic neurological syndrome. Due to the tumor’s remote effects, it causes a series of neurological symptoms, mainly affecting the limbic system, diencephalon, and brainstem. The clinical manifestations of encephalitis, which typically manifest before the tumor is diagnosed, vary depending on the location and extent of the lesion (1). When rapidly progressing dementia, extrapyramidal dysfunction, hallucination, and psychiatric symptoms occur, the diagnosis of the disease requires differentiation from CJD. Previous meta-analysis studies suggested that the sensitivity and specificity of the 14-3–3 protein for the diagnosis of CJD were 92% and 80%, respectively (7). However, this biomarker has also been increasingly detected in non-prion-related rapid progressive dementia in recent years. Kong et al. found that CSF 14-3–3 protein was discovered in 23.3% of non-prion rapid progressive dementia patients, the most common being AE, with a positive rate of 22.9% (3). Bastiaansen et al. indicated that the positivity rate for 14-3–3 protein in CSF was 19% in patients diagnosed with AE (4). In addition, AE-associated antineuronal antibodies have also been found in CJD (3, 4). Based on previous research reports, neuronal antibodies co-occurring with 14-3–3 protein have exclusively targeted neuronal surface antigens, such as anti-NMDAR antibody, CASPR2 antibody, GlyR antibody, LGI1 antibody, GABA_B_R antibody and GluR2 antibody (2, 4, 8, 9). The anti-Ma2 antibody, an autoantibody targeting intracellular antigens, has not been reported as far as we know. However, the biological mechanism of this coexistence phenomenon remains unclear. 14-3–3 protein is a highly conserved family of cellular proteins abundantly expressed in the brain and plays a key role in regulating central physiological pathways (10). It is involved in signaling pathway conduction, cell growth, and apoptosis and, like other CSF markers, represents neuronal damage (4). Moreover, 14-3–3 protein has potential roles in the initiation and progression of cancer. Kong et al. discovered that 14-3–3 protein can be present in the CSF of patients diagnosed with central nervous system tumors (3). Anti-Ma2 encephalitis is also an AE significantly associated with the cancer. Therefore, positive CSF 14-3–3 protein likely reflects immune-mediated neuronal damage driven by anti-Ma2 antibody. This antibody target intracellular neuronal antigens, triggering cytotoxic T-cell immunity (11), which induces widespread neuronal destruction with subsequent 14-3–3 protein release. Although 14-3–3 positivity has been reported in autoimmune encephalitides targeting surface antigens, this study provides the first evidence of this phenomenon in encephalitis associated with intracellular antigens (anti-Ma2 encephalitis), thereby broadening the disease spectrum of antibody coexistence syndromes.

Following discharge, the patient returned to his hometown for continued management. Telephone follow-ups at 1, 3, and 6 months confirmed preserved neurological function without evidence of disease relapse. However, the patient died from severe pneumonia (unrelated to neurological deterioration) prior to planned re-evaluation, which prevented formal longitudinal biomarker and imaging assessments. The family declined autopsy due to personal and cultural considerations. Although this limits histopathological correlation of neuronal injury mechanisms, we emphasize that the acute-phase diagnostic evidence remains unequivocal:1) The high titer of anti-Ma2 antibodies confirmed in CSF and serum; 2) Exclusion of prion disease via RT-QuIC and PRNP gene analysis; A large cohort study demonstrated that the analysis of CSF samples from individuals suspected of having CJD using the RT-QuIC could identify prions with a specificity rate of up to 100% and a sensitivity rate of up to 95% (9). Furthermore, Rossi et al. reported 256 cases of CJD and discovered that less than 5% of patients with CJD developed serum antibodies against neuronal antigens; however, these antibodies were present only at low concentrations (8). 3) The presence of bilateral temporal lobe hyperintensity on FLAIR/DWI MRI (distinct from the cortical “ribboning” or basal ganglia hyperintensity typically associated with CJD) (12); 4) Objective neurological improvement post-immunotherapy. Consequently, detection of high-titer antineuronal antibodies should prompt aggressive pursuit of treatable autoimmune etiologies, overriding overreliance on 14-3–3 protein. In addition, the clinical significance of the coexistence of anti-Ma2 antibody and 14-3–3 protein is unclear, we speculate that this may indicate a higher severity of the disease and extensive neuronal damage. This warrants validation in future studies.

## Data Availability

The original contributions presented in the study are included in the article/Supplementary Material. Further inquiries can be directed to the corresponding author/s.

## References

[1] Du Rusquec P Peyre A Toulgoat F Honnorat J Raimbourg J . Fatal anti-ma2 encephalitis related to treatment of Malignant pleural mesothelioma with a combination of anti-programmed death 1 and anti-cytotoxic T-lymphocyte associated protein 4 antibodies. J Thorac Oncol. (2019) 14:e174–6. doi: 10.1016/j.jtho.2019.03.017, PMID: 31345342

[2] Zuhorn F Hübenthal A Rogalewski A Dogan Onugoren M Glatzel M Bien CG . Creutzfeldt-Jakob disease mimicking autoimmune encephalitis with CASPR2 antibodies. BMC Neurol. (2014) 14:227. doi: 10.1186/s12883-014-0227-7, PMID: 25434587 PMC4255969

[3] Kong Y Chen Z Shi Q Zuo Y Zhang J . Clinical correlates of cerebrospinal fluid 14-3–3 protein in non-prion rapid progressive dementia. J Alzheimers Dis. (2023) 91:263–72. doi: 10.3233/jad-220718, PMID: 36404548

[4] Bastiaansen AEM van Steenhoven RW de Bruijn M Crijnen YS van Sonderen A van Coevorden-Hameete MH . Autoimmune encephalitis resembling dementia syndromes. Neurol Neuroimmunol Neuroinflamm. (2021) 8:e1039. doi: 10.1212/nxi.0000000000001039, PMID: 34341093 PMC8362342

[5] Wang BJ Wang CJ Zeng ZL Yang Y Guo SG . Lower dosages of rituximab used successfully in the treatment of anti-NMDA receptor encephalitis without tumour. J Neurol Sci. (2017) 377:127–32. doi: 10.1016/j.jns.2017.04.007, PMID: 28477682

[6] Deng B Yu H Liu X Yu X Zhang X Li X . Reduced dosage rituximab in the treatment of anti-N-methyl-d-aspartate receptor encephalitis: An observation study in Chinese patients. J Neuroimmunol. (2019) 330:81–6. doi: 10.1016/j.jneuroim.2019.02.008, PMID: 30851542

[7] Muayqil T Gronseth G Camicioli R . Evidence-based guideline: diagnostic accuracy of CSF 14-3–3 protein in sporadic Creutzfeldt-Jakob disease: report of the guideline development subcommittee of the American Academy of Neurology. Neurology. (2012) 79:1499–506. doi: 10.1212/WNL.0b013e31826d5fc3, PMID: 22993290 PMC3525296

[8] Rossi M Mead S Collinge J Rudge P Vincent A . Neuronal antibodies in patients with suspected or confirmed sporadic Creutzfeldt-Jakob disease. J Neurol Neurosurg Psychiatry. (2015) 86:692–4. doi: 10.1136/jnnp-2014-308695, PMID: 25246643 PMC4453627

[9] Foutz A Appleby BS Hamlin C Liu X Yang S Cohen Y . Diagnostic and prognostic value of human prion detection in cerebrospinal fluid. Ann Neurol. (2017) 81:79–92. doi: 10.1002/ana.24833, PMID: 27893164 PMC5266667

[10] Tzivion G Gupta VS Kaplun L Balan V . 14-3–3 proteins as potential oncogenes. Semin Cancer Biol. (2006) 16:203–13. doi: 10.1016/j.semcancer.2006.03.004, PMID: 16725345

[11] Bien CG Vincent A Barnett MH Becker AJ Blümcke I Graus F . Immunopathology of autoantibody-associated encephalitides: clues for pathogenesis. Brain. (2012) 135:1622–38. doi: 10.1093/brain/aws082, PMID: 22539258

[12] Bizzi A Pascuzzo R Blevins J Grisoli M Lodi R Moscatelli MEM . Evaluation of a new criterion for detecting prion disease with diffusion magnetic resonance imaging. JAMA Neurol. (2020) 77:1141–9. doi: 10.1001/jamaneurol.2020.1319, PMID: 32478816 PMC7265127

